# HAZARDOUS WASTE: TVA Spill’s Chemical Legacy

**DOI:** 10.1289/ehp.117-a346a

**Published:** 2009-08

**Authors:** Rhitu Chatterjee

**Affiliations:** **Rhitu Chatterjee** is a freelance journalist based in Bangalore, India. She covers science and environmental stories for various print, radio, and online news outlets including *Science* and National Public Radio

When the holding pond at the Tennessee Valley Authority’s (TVA) Kingston coal-burning power plant broke on the morning of 22 December 2008, it released more than 5.4 million yd^3^—some 50 years’ worth of accumulation—of coal ash slurry into the neighboring lands and the Emory River. High levels of arsenic and mercury in the spilled slurry could pose serious environmental and human health risks, according to the first peer-reviewed assessment of the chemical contamination from this spill, published 1 August 2009 in *Environmental Science & Technology*.

Scientists have studied the chemical composition of coal ash for decades. They have a general understanding of how coal ash constituents travel through the environment but lack data on how spills translate into long-term human and ecologic health hazards. So environmental scientists Avner Vengosh and Laura Ruhl of the Nicholas School of the Environment at Duke University visited Tennessee in the months after the spill to collect samples of coal ash slurry, sediments, and water from various sites along the Emory and Clinch Rivers, which flow into the Tennessee River, the primary source of drinking water for some 410,000 Tennesseans.

Data reported by the Tennessee Department of Environment and Conservation showed the ash contained elevated levels of arsenic and mercury. With time, wrote Vengosh and colleagues, as the mass of slurry begins to dry, these toxic elements might become airborne and pose a health threat to local communities. However, investigating whether this is indeed happening would require long-term air monitoring, says Vengosh.

Trace elements including arsenic, selenium, lithium, and boron were measured at elevated levels in a tributary of the Emory that was dammed by the spill and turned into a standing pond. The concentration of dissolved arsenic in this pond was as high as 86 μg/L, whereas unaffected upstream waters contained 0.1–0.4 μg/L. Concentrations of these elements were significantly lower at the downstream Emory and Clinch River sites but were above background concentrations, suggesting that leaching of these toxicants was balanced by massive river dilution.

Although dilution improved the water quality, it could not control the deposition of ash in the river sediments. The mercury levels in sediments a couple miles downstream of the spill were almost as high as those in the coal ash itself, around 92–130 μg/kg. Anaerobic bacteria living in these sediments could convert this mercury to its more bioaccumulative and toxic form, methylmercury.

Frank Huggins, an environmental chemist at the University of Kentucky, isn’t convinced we yet know the real risk to humans and wildlife. The authors use the word “potential,” he notes. “Sure there is always a lot of potential, but . . . the actual risk is a much more difficult topic to address.” Huggins is just beginning to analyze some samples from the spill site for the TVA. He says complementary and longer-term analyses of the elements, their speciation, and how they behave over time and under different physical conditions in sediments and water will reveal the true hazards.

Ecotoxicologist William Hopkins of Virginia Polytechnic Institute and State University says future studies will need to investigate which of the trace elements measured by Vengosh and colleagues are bioavailable to biota in the area as well as the severity of any effects resulting from exposure. Some such investigations have already begun. The TVA recently hired Hopkins as a consultant to begin looking at issues of bioaccumulation and toxicity. Similarly, ecophysiologist Shea Tuberty of Appalachian State University is studying how the spill has affected levels of selenium and other elements in fish tissue, and how that in turn affects fish populations. His preliminary work has established baseline selenium levels in local fish populations that are already close to levels that cause reproductive failure in most aquatic species—possibly the result of decades of selenium leaching from the holding ponds, he says.

Vengosh and colleagues agree that much more research is needed to evaluate the long-term environmental and human health effects of the spill. With the support of a new project funded by the National Science Foundation, the Duke team will continue to investigate these effects.

